# Efficacy and safety of concurrent anti-Cancer and anti-tuberculosis chemotherapy in Cancer patients with active *Mycobacterium tuberculosis*: a retrospective study

**DOI:** 10.1186/s12885-018-4889-1

**Published:** 2018-10-12

**Authors:** Tomonori Hirashima, Yoshitaka Tamura, Yuki Han, Shoji Hashimoto, Ayako Tanaka, Takayuki Shiroyama, Naoko Morishita, Hidekazu Suzuki, Norio Okamoto, Shinobu Akada, Makoto Fujishima, Yoshihisa Kadota, Kazuya Sakata, Akiko Nishitani, Satoru Miyazaki, Takayuki Nagai

**Affiliations:** 1 Department of Thoracic Oncology, Osaka Habikino Medical Center, 3-7-1 Habikino, Habikino City, Osaka 583-8588 Japan; 2 Departments of Clinical Laboratory, Osaka Habikino Medical Center, 3-7-1 Habikino, Habikino City, Osaka 583-8588 Japan; 3 Departments of Infectious Diseases, Osaka Habikino Medical Center, 3-7-1 Habikino, Habikino City, Osaka 583-8588 Japan; 4 Departments of Gynecology, Osaka Habikino Medical Center, 3-7-1 Habikino, Habikino City, Osaka 583-8588 Japan; 5 Departments of Breast Surgery, Osaka Habikino Medical Center, 3-7-1 Habikino, Habikino City, Osaka 583-8588 Japan; 6 Departments of Thoracic Surgery, Osaka Habikino Medical Center, 3-7-1 Habikino, Habikino City, Osaka 583-8588 Japan; 7 Departments of Gastroenterological Surgery, Osaka Habikino Medical Center, 3-7-1 Habikino, Habikino City, Osaka 583-8588 Japan

**Keywords:** Concurrent chemotherapy, Tuberculosis, Breast cancer, Colorectal cancer, Efficacy, Gastric cancer, Lung cancer, Safety

## Abstract

**Background:**

In our previous study, colorectal cancer (CRC) patients with active *Mycobacterium tuberculosis* (*MTB*) tolerated concurrent anti-cancer chemotherapy (anti-CCT) and anti-*MTB* chemotherapy. In this study, we retrospectively confirmed the efficacy and safety of concurrent chemotherapy in a greater number of patients with different types of malignancies.

**Methods:**

We enrolled 30 patients who were treated concurrently with anti-CCT and anti-*MTB* regimens between January 2006 and February 2016. Cancer and *MTB* treatments were administered according to the approved guidelines.

**Results:**

Patient demographics included: men/woman: 24/6; median age: 66.5 years; Eastern Cooperative Oncology Group performance status 0–1/2/3–4: 24/4/2; Stage IIB–IIIC/IV/recurrence: 6/22/2; lung cancer (LC)/CRC/other: 15/10/5; and *MTB* diagnosis (before or during anti-CCT): 20/10 (LC: 8/7; CRC: 8/2; other: 4/1). For anti-CCT, 23 patients received two cytotoxic agents with or without targeted agents and 7 patients received a single cytotoxic or targeted agent. The overall response rate was 36.7%. Regarding anti-*MTB* chemotherapy, 22 patients received a daily drug combination containing isoniazid, rifampicin, and ethambutol, plus pyrazinamide in 15 of the 22 patients, followed by daily isoniazid and rifampicin; the remaining 8 patients received other combinations. Hematological adverse events of Grade ≥ 3 were observed in 19 (67.9%) of 28 patients; laboratory data were lost for the remaining 2. Grade 3 lymphopenia and higher were significantly more frequent in LC compared to other malignancies (*P* < 0.005). Non-hematological adverse events of Grade ≥ 3 were observed in 5 (16.7%) of 30 patients. One CRC patient experienced Grade 3 hemoptysis and another 2 experienced Grade 3 anaphylaxis. One patient with cholangiocellular carcinoma and gastric cancer experienced Grade 3 pseudomembranous colitis as a result of a *Clostridium difficile* infection. One patient (3.3%) died of pemetrexed-induced pneumonitis. The success of the anti-*MTB* chemotherapy was 70.0%. There were no *MTB*-related treatment failures. The median overall survival (months, 95.0% confidence interval) was 10.5 (8.7–36.7), 8.7 (4.7–10.0), 36.7 (minimum 2.2), and 14.4 (minimum 9.6) for all patients combined, LC, CRC, and Other malignancies, respectively. LC patients experienced delayed *MTB* diagnosis and shorter overall survival.

**Conclusions:**

Concurrent chemotherapy is effective and safe for treating cancer patients with active *MTB*.

## Background

*Mycobacterium tuberculosis* (*MTB*) represents the leading cause of death from an infectious disease worldwide [1], with the majority of cases occurring in Asia (61.0%) and Africa (26.0%). Incidence and mortality rates are noticeably higher in Japan than other developed countries [1].

Although active *MTB* infections may be present in cancer patients, our previous preliminarily report [2] is the only study to discuss the clinical course and chemotherapy outcomes of concurrent anti-cancer chemotherapy (anti-CCT) and anti-*MTB* chemotherapy, revealing that patients with metastatic colorectal cancer (CRC) and active *MTB* could safely and effectively continue anti-CCT, and achieve comparable survival to those without the infection, upon receiving appropriate *MTB* treatment [2].

In this study, we retrospectively examined the clinical course and chemotherapy outcomes of a larger number of patients treated concurrently with anti-CCT and anti-*MTB* chemotherapy.

## Methods

### Study approval

The present retrospective study was approved by the Institutional Review Board (IRB) of the Osaka Habikino Medical center on 30 January 2017 (approval number: 808–1). The board waived the requirement for informed written consent due to the anonymous nature of the data, and no risk of exposure to subjects.

### Patient selection

We enrolled 30 cancer patients with active *MTB* who were treated concurrently with anti-CCT and anti-*MTB* chemotherapy at our institution between January 1, 2006 and February 29, 2016. The 6 metastatic CRC patients with active *MTB* from our previous study [2] were also included.

### Clinical review

As described previously, [2] the clinical history of eligible patients was retrospectively reviewed. We collected baseline demographic data and anti-CCT data, which were also collected from clinical records or pharmacy database. Complete history and physical examinations; surgical reports; findings of flexible bronchoscopy, colonoscopy, and esophagogastroduodenoscopy; imaging investigations; pathological reports, and blood test results were available for all patients at the time of anti-*MTB* chemotherapy.

### *MTB* diagnosis

As described previously [2], *MTB* diagnosis was performed by smears and cultures of various patients’ specimens or chest computed tomography (CT) image in patients without microbiological evaluation. The method of choice for confirming *MTB* infection was Ziehl-Neelsen staining of sputum smear samples [3]. Polymerase chain reaction (PCR) or loop-mediated isothermal amplification (LAMP) [4] was performed for patients with positive sputum smears to distinguish *MTB* from other mycobacteria. If the sputum smears were negative or specimens other than sputum were obtained, the diagnosis of *MTB* was confirmed by culturing mycobacterial organisms. Drug sensitivity was determined for all cases with positive culture. Liquid media with the Mycobacteria Growth Indicator Tube (MGIT) [5] and solid media with the Ogawa-Kudoh method [6] were both used for culturing mycobacteria. Drug sensitivity was determined for all cases. Quantitative drug susceptibility testing for *MTB* was performed using the MTB-I® (Kyokuto Pharmaceutical Industrial Co., Ltd., Tokyo, Japan) modified Minimum Inhibitory Concentration method [7].

### Treatment of *MTB* infection

As described previously [2], in cancer patients with active *MTB*, appropriate anti-*MTB* agents were administered for approximately 1.5 months prior to anti-CCT, according to the American Thoracic Society and Infectious Diseases Society of America guidelines [8] A multi-drug resistant *MTB*, based on the sensitivity test, negated the initiation of anti-CCT. Since *MTB* patients with severe complications often require longer courses of treatment than those without such complications, patients treated with anti-CCT also received long-term treatment for *MTB.* Thus, the patients received 2 months of isoniazid (H), rifampicin (R), ethambutol (E), and pyrazinamide (Z), followed by HR for 7 months, or 6 months of HRE, followed by HR for 6 months as standard anti-*MTB* chemotherapy. The majority of patients who could not be treated with standard anti-*MTB* chemotherapy, due to side effects or drug resistance, were treated with a levofloxacin (X)-based regimen.

### Follow-up *MTB* culture

After commencing anti-*MTB* chemotherapy, sputum specimens were cultured every other week for the first 3 months. Once two consecutive sputum cultures were negative, cultures were done monthly until the course of *MTB* treatment was completed.

### Definition of *MTB* treatment outcomes

*MTB* treatment outcomes were based on the World Health Organization’s definitions [9]. Treatment success included “cure” and “treatment completed”. A “cured” patient was defined as one who had completed the planned treatment and had two consecutive negative cultures. Patients with treatment failure had positive cultures ≥5 months into *MTB* treatment.

### Cancer staging and treatment

Cancer staging and treatment were performed according to the guidelines of the respective lung cancer (LC) [10], CRC [11], gastric cancer (GC) [12], breast cancer, [13], and cholangiocellular carcinoma (CCA) [14] societies.

The policy of our institution was to commence anti-CCT immediately if patients were sensitive to anti-*MTB* agents. However, if patients were resistant or had experienced intolerable side effects while awaiting sensitivity test, anti-CCT was suspended until a decision could be reached concerning the appropriate drug combination and duration of anti-*MTB* chemotherapy. Additionally, when *MTB* cultures could not be obtained, due to a number of reasons, or the physician could not await the results of the sensitivity test due to rapid tumor progression, anti-CCT was commenced, following informed patient consent.

### Assessment of anti-cancer chemotherapy outcomes

Patients’ best response to chemotherapy was collected from the records of weekly meetings at our institution and clinical summaries. Based on the Response Evaluation Criteria in Solid Tumors [15], the responses of tumors to cytotoxic agents were categorized as complete or partial responses and stable or progressive disease. Tumor responses that could not be assessed were recorded as “not evaluable”. Adverse events were graded according to the National Cancer Institute Common Toxicity Criteria, version 3.0 [16].

### Statistical analyses

Overall survival (OS) was measured from the date of commencing concurrent chemotherapy (for both cancer and *MTB*) to the date of death or last follow-up (February 28, 2017). OS rates were estimated using the Kaplan-Meier method [17]. The duration of concurrent chemotherapy was defined as the time from commencing concurrent chemotherapy to the end date. All statistical analyses were conducted using R statistical software (version 3.2.0). Patient background data were analyzed using chi-square and Fisher’s exact test for categorical variables. A *P* < 0.05 was considered statistically significant.

## Results

### Patient demographics

In total, 30 cancer patients with active *MTB* who were treated concurrently with anti-CCT and anti-*MTB* chemotherapy between January 1, 2006 and February 29, 2016 at our institution, were enrolled in this study. Fifteen patients were diagnosed with LC (non-small cell LC [NSCLC, *n* = 10] and small cell LC [SCLC, *n* = 5]), 10 patients were diagnosed with CRC (rectal cancer [*n* = 7], sigmoid cancer [*n* = 2], and transverse colon cancer [*n* = 1]), and 5 patients were diagnosed with other malignancies (GC [*n* = 1], Breast Cancer [*n* = 1], LC with GC [*n* = 1], CRC with GC [*n* = 1], and CCA with GC [*n* = 1]). The patient demographics are summarized in Table 1. No significant differences in patient demographics were observed among LC, CRC, and other malignancy groups. However, LC patients tended to be more frequently diagnosed with active *MTB* during anti-CCT in comparison to those with CRC or other malignancy.

**Table 1:** Patient Characteristics

Characteristic	Patients			
	All	LC	CRC	Other
	(*n* = 30)	(*n* = 15)	(*n* = 10)	(*n* = 5)
Sex, n (%)				
M	24 (80.0)	12 (80.0)	8 (80.0)	4 (80.0)
F	6 (20.0)	3 (20.0)	2 (20.0)	1 (20.0)
Age (years), median (range)	66.5 (39–79)	68.0 (43–79)	62.5 (43–75)	56.0 (39–75)
ECOG PS, n (%)				
0–1	24 (80.0)	12 (80.0)	8 (80.0)	4 (80.0)
2	4 (13.3)	3 (20.0)	0 (0.0)	1 (20.0)
3	2 (6.7)	0 (0.0)	2 (20.0)	0 (0.0)
Stage, n (%)				
IIB-IIIA	3 (10.0)	2 (13.3)	0 (0.0)	1 (20.0)
IIIB-ΙΙΙC	3 (10.0)	2 (13.3)	0 (0.0)	1 (20.0)
IV	22 (73.3)	10 (66.7)	9 (90.0)	3 (60.0)
Postoperative recurrence, n (%)	2 (6.7)	1 (6.7)	1 (10.0)	0 (0.0)
Line of cancer chemotherapy at the commencement of concurrent chemotherapy, n (%)				
Adjuvant	1 (3.3)	0 (0.0)	0 (0.0)	1 (20.0)
First	21 (70.0)	9 (60.0)	8 (80.0)	4 (80.0)
Second	3 (10.0)	3 (20.0)	0 (0.0)	0 (0.0)
Third or higher	5 (16.7)	3 (20.0)	2 (20.0)	0 (0.0)
Diabetes mellitus, n (%)				
Y	7 (20.7)	3 (20.0)	2 (20.0)	2 (40.0)
N	23 (79.3)	12 (80.0)	8 (80.0)	3 (60.0)
*MTB* diagnosis, n (%)				
Before anti-CCT	20 (66.7)	8 (53.3)	8 (80.0)	4 (80.0)
During anti-CCT	10 (33.3)	7 (46.7)	2 (20.0)	1 (20.0)

*Abbreviations CCT* cancer chemotherapy, *CRC* colorectal cancer, *ECOG* Eastern Cooperative Oncology Group, *F* female, *LC* lung cancer, *M* male, *MTB Mycobacterium tuberculosis, N* no, *PS* performance status, *Y* yes

### Chest CT imaging

**Fig. 1:**
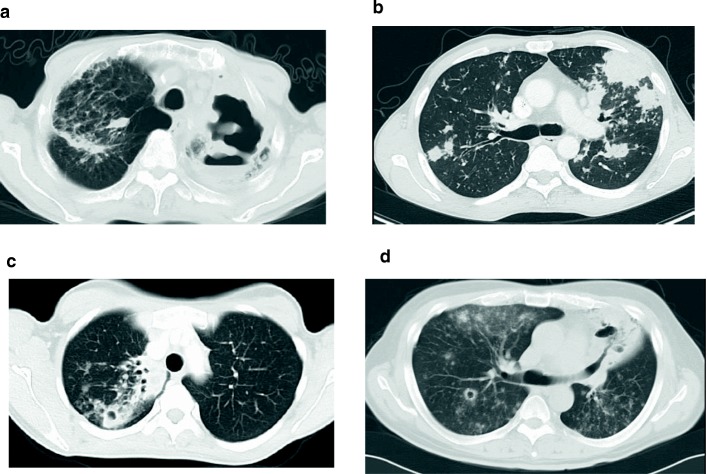
Thoracic computed tomography findings in 4 cancer patients with active *Mycobacterium tuberculosis*. **a** Non-small cell lung cancer patient with a thick wall cavity in the left lung with an infiltration shadow in the right upper lobe. **b** Colorectal cancer patient with multiple nodules in both lungs. **c** Breast cancer patient with a small cavity with an infiltration shadow in the right upper lobe. **d** Non-small cell lung cancer patient with multiple small nodules with partial patty follicular spot in the both lungs and cavity formation in the S6 segment of the right lung and lingular segment of the left lung

Thoracic CT findings at the time of *MTB* diagnosis is presented in Fig. 1 (a-d). These CT images present a combination of thick and thin walled lung cavities, infiltration shadows, and multiple nodules. There were no consistent findings on CT imaging based on the type of malignancy.

### Bacteriological examinations

The findings of bacteriological examinations are summarized in Table 2. Twenty (66.7%) patients had *MTB*-positive sputum smears. In 25 (83.3%) cultures were positive for *MTB* and negative cultures were reported in 5 (16.7%). Cultures were negative for *MTB* in 3 LC and 2 CRC patients. In the former, active *MTB* was diagnosed by PCR of sputum in 2 cases and LAMP of sputum in 1, alongside CT results, while in the latter, the diagnosis was confirmed by PCR of sputum in one, and the results of the CT scan and clinical course in the other.

**Table 2:** Bacteriological Examinations

Characteristic	Patients			
	All	LC	CRC	Other
	(*n* = 30)	(*n* = 15)	(*n* = 10)	(*n* = 5)
Sputum smear, n (%)				
Negative	10 (33.3)	5 (33.3)	3 (30.0)	2 (40.0)
Positive	20 (66.7)	10 (66.7)	7 (70.0)	3 (60.0)
PCR/LAMP, n (%)				
Negative	1 (3.3)	1 (6.7)	0 (0.0)	0 (0.0)
Positive	15 (50.0)	10 (66.7)	3 (30.0)	2 (40.0)
Unknown	14 (46.7)	4 (26.7)	7 (70.0)	3 (60.0)
Culture, n (%)				
Negative	5 (16.7)	3 (20.0)	2 (20.0)	0 (0.0)
Positive	25 (83.3)	12 (80.0)	8 (80.0)	5 (100.0)
Sensitivity to anti-*MTB* agents, n (%)				
Sensitive	22 (88.0)	12 (100.0)	6 (75.0)	4 (80.0)
Resistant	2 (8.0)^a^	0 (0.0)	2 (25.0)	0 (0.0)
Unknown	1 (4.0)^b^	0 (0.0)	0 (0.0)	1 (20.0)^b^
Time until the result of Bacteriological Examination come out				
	Overall	Ogawa-Kudoh		MGIT
Time to *MTB*-positive culture (days), mean ± SD, n	20.9 ± 12.4 (*n*=23^c^)	37.6 ± 8.4 (*n* = 5)		16.2 ± 8.7^*^ (*n* = 18)
Time to sensitivity testing (days), mean ± SD, n	12.6 ± 3.5 (*n*=22^d^)			

*Abbreviations CRC* colorectal cancer, *LAMP* loop-mediated isothermal amplification, *LC* lung cancer, *MGIT* Mycobacteria Growth Inhibitor Tube, *MTB* = *Mycobacterium tuberculosis*, *PCR* polymerase chain reaction, *NTM Nontuberculous Mycobacteriosis*

^*^P < 0.0005

^a^One *MTB* strain was resistant to isoniazid and streptomycin, another was isoniazid and pyrazinamide

^b^One specimen with *MTB*-positive cultures and concomitant NTM was not used for sensitivity testing

^c^In two specimens, measure of the time to *MTB*-positive cultures were not possible because the culture specimens were obtained in other institutes

^d^Twenty-two cultures except one which was concomitant NTM and two which was obtained in other institute

In the 25 cases positive for *MTB*, 22 (88.0%) were sensitive, 2 (8.0%) were resistant (one *MTB* strain was resistant to H and streptomycin, another to H and Z), and 1 (4.0%) was not tested for sensitivity because of concomitant *Nontuberculous Mycobacteriosis*. In 2 of 25 patients with *MTB*-positive cultures, the time to *MTB*-positive culture could not be confirmed because the culture specimens were obtained in other institutes. Therefore, the time to *MTB*-positive culture in 23 patients was demonstrated by MGIT (*n* = 18) and Ogawa-Kudoh method (*n* = 5). The time (days: mean ± SD) to *MTB*-positive cultures was significantly shorter for MGIT than for the Ogawa-Kudoh method (16.2 ± 8.7 vs 37.6 ± 8.4; *P* < 0.0005). Furthermore, the time (days: mean ± SD) to sensitivity testing in 22 patients except one with concomitant *Nontuberculous Mycobacteriosi* was 12.6 ± 3.5.

### *MTB* treatment until concurrent chemotherapy

Of the 30 patients enrolled in this study, 4 received X-based regimen from the start of *MTB* treatment due to resistant to INH (*n* = 2), past history of RFP-induced systemic eruption (*n* = 1), and preventing the drug interaction between erlotinib and RFP via CYP3A4 (*n* = 1).

In 2 of 4 patients the X-based regimen was changed to a different regimen due to renal dysfunction in one and liver dysfunction in the other.

In 4 patients the planned HREZ or HRE regimen was changed to X-based regimen due to drug eruption (*n* = 1), drug eruption and liver dysfunction (*n* = 1), thrombocytopenia (*n* = 1), and preventing the drug interaction between Paclitaxel and RFP via CYP3A4 and CYP2C8 (*n* = 1). Subsequently, 8 patients received concurrently X-based regimens and anti-CCT. The remaining 22 patients received concurrent HREZ or HRE and anti-CCT as planned, without many adverse events except in one patient who experienced a paradoxical response on day 23 after starting HREZ and was kept on HREZ while taking prednisolone. Thus, until concurrent chemotherapy, 6 (20.0%) of the 30 patients experienced adverse events with *MTB* treatment alone, and in 5 the planned *MTB* treatment was changed to other regimens.

### Outcomes of concurrent chemotherapy

Details of the initial concurrent anti-CCT regimens are listed in Table 3. Twenty-three patients (76.7%) received intensive anti-CCT regimens with or without targeted agents, 5 patients (16.7%) received a single cytotoxic agent, and 2 patients (6.7%) received a single targeted agent. The overall response rates (ORRs) were 36.7, 33.3 40.0, and 40.0% for all patients combined, LC patients, CRC patients, and patients with other malignancies, respectively. In 15 LC patients, all 5 patients with SCLC received intensive cytotoxic treatment (carboplatin plus etoposide) as first line chemotherapy (Table 3) with a high response rate (80%). Of 10 NSCLC patients, 4 (40%) received platinum doublets as intensive first regimen, and one of them achieved partial response. In the remaining 6 patients (60%), one received platinum doublets, three were administered single cytotoxic agent, and two received erlotinib as re-challenge regimen. They received those regimens as second-line or later anti-CTT and there was no responder.

**Table 3:** Outcomes of Anti-Cancer Chemotherapy (Anti-CCT) and Anti-*MTB* Chemotherapy

Characteristic	Patients			
	All	LC (SCLC/NSCLC)	CRC	Other
	(*n* = 30)	(*n* = 15, 5/10)	(*n* = 10)	(*n* = 5)
Initial concurrent anti-CCT regimen, n				
Intensive cytotoxic regimen and targeted agent^a^	4	0	3	1
Intensive cytotoxic regimen^b^	19	10 (5/5)	6	3
Single targeted agent^c^	2	2 (0/2)	0	0
Single cytotoxic agent^d^	5	3 (0/3)	1	1
Best response on anti-CCT after commencing concurrent chemotherapy, n				
CR	1	0	1	0
PR	10	5 (4/1)	3	2
SD	5	2 (0/2)	3	0
PD	6	4 (0/4)	2	0
NE	8	4 (1/3)	1	3
ORR (%)^e^	36.7	33.3 (80.0/10.0)	40.0	40.0
Main anti-*MTB* chemotherapy, n				
2HREZ/7HR^f^	15	8 (3/5)	4	3
6HRE/6HR^g^	7	2 (0/2)	4	1
Levofloxacin-based	8	5 (2/3)	2	1
Duration of anti-*MTB* treatment (days), median (range)	275.0 (72–637)	274.0 (90–469) [274 (183–469)/255 (90–310)]	259.0 (72–539)	368.0 (273–637)
Duration of concurrent chemotherapy (days), median (range)	157.5 (13–408)	117.0 (20–245) [93 (20–207)/121 (48–245)]	168.5 (13–408)	155.0 (51–376)
*MTB* treatment outcomes, n (%)	All	LC	CRC	Other
Cured	20 (66.7)	10 (66.7)	6 (60.0)	4 (80.0)
Completed	1 (3.3)	0	1 (10.0)	0
Died	9 (30)	5 (33.3)	3 (30.0)	1 (20.0)
Failed	0	0	0	0
Not evaluated	0	0	0	0
Success (%)^h^	70.0	66.7	70.0	80.0

*Abbreviations CI* confidence interval, *CR* complete response, *CRC* colorectal cancer, *E* ethambutol, *EGFR-TKI* epidermal growth factor receptor-tyrosine kinase inhibitor, *H* isoniazid, *LC* lung cancer, *MTB Mycobacterium tuberculosis, NE* not evaluable, *ORR* overall response rate, *PD* progressive disease, *PR* partial response, *R* rifampicin, *SD* stable disease, *Z* pyrazinamide

^a^Two cytotoxic agents combined with targeted therapy (bevacizumab or trastuzumab)

^b^Two cytoxic agents

^c^Erlotinib

^d^Single cytoxic agent (S-1, vinorelbine, or pemetrexed)

^e^CR + PR

^f^Daily drug combination containing HREZ for 2 months, followed by daily HR for 7 months

^g^Daily drug combination containing HRE for 6 months, followed by HR for 6 months

^h^Cured + completed

The main anti-*MTB* regimens were as follows: HREZ/HR (*n* = 15 patients; LC [*n* = 8], CRC [*n* = 4], other [*n* = 3]); HRE/HR (*n* = 7 patients; LC [*n* = 2], CRC [*n* = 4], other [*n* = 1]); and X-based (*n* = 8 patients; LC [*n* = 5]. and CRC [*n* = 2], other [*n* = 1]). The median duration (range) of *MTB* treatment was 275.0 (72–637), 274.0 (90–469), 259.0 (72–539), and 368.0 (273–637) days for all patients combined, those with LC, CRC, and other malignancies, respectively. The success of anti-*MTB* chemotherapy in each of the above groups was 70.0, 66.7, 70.0, and 80.0%, respectively. There were no *MTB*-related treatment failures. The median duration (range) of concurrent chemotherapy was 157.5 (13–408), 117.0 (20–245), 168.5 (13–408), and 155.0 (51–376) days for all patients combined, those with LC, CRC, and other malignancies, respectively.

In 20 (66.7%) patients *MTB* was cured, one (3.3%) completed the course for *MTB* treatment, and 9 (30%) died while receiving *MTB* treatment. Among the 9 patients who died, one died of pemetrexed-induced pneumonitis and 8 died of cancer.

In 2 CRC patients with performance status of 3, anti-CCT was started because of patients’ insistence and after the approval of the Cancer Board. In one of these patients, *MTB* was resistant to H and Z. Hence, X + Streptomycin was administered, alongside Folnic acid, fluorouracil and irinotecan for 1 cycle. The patient died 54 days after treatment withdrawal. Regular *MTB* cultures performed every other week were negative for six consecutive times. The other patient received modified regimen of Folinic acid, fluorouracil and oxaliplatin for 4 cycles and died 57 days after treatment withdrawal.

Epidermal growth factor receptor sensitive mutation was found in 2 *MTB*-positive patients with lung adenocarcinoma. They received erlotinib as re-challenge regimen alongside HRE regimen in one patient and X-based regimen without rifampicin in the other. Both died because of cancer progression, 140 and 90 days after the initiation of erlotinib, respectively. Regular *MTB* cultures were performed for both patients every other week. Cultures were negative in each patient two and three consecutive times, respectively. None of the 30 patients enrolled in this study experienced recurrence of *MTB* through their clinical course.

### Adverse events of concurrent chemotherapy

Hematological adverse events of Grade ≥ 3 were observed in 19 (67.9%) of 28 patients; the laboratory data for 2 patients were lost. Non-hematological adverse events of Grade ≥ 3 were observed in 5 (17.9%) of 28 patients.

Number of cases in concurrent chemotherapy-related adverse events are shown in Table 4. There were no significant differences in the occurrence of adverse events between different cancer types, except for lymphopenia and neutropenia. Grade 3 lymphopenia and higher were significantly more frequent in LC compared to other malignancies (*P* < 0.005). Grade 3 Neutropenia and higher tended to be more frequent in LC compared to other malignancies. In non-hematological adverse events, Grade 1–2 liver dysfunction was frequently observed in each malignancies.

**Table 4:** Concurrent Chemotherapy-Related Adverse Events

NCI-CTC^a^ (Grade)	Patients							
	All		LC		CRC		Other	
	(*n* = 28)		(*n* = 13^b^)		(*n* = 10)		(*n* = 5)	
Adverse event	1–2	≥3	1–2	≥3	1–2	≥3	1–2	≥3
Hematological toxicity, number of cases								
Leukocytopenia	7	8	4	6	3	1	2	1
Neutropenia	5	14	1	9	2	3	2	2
Anemia	20	2	9	2	8	0	3	0
Thrombocytopenia	3	3	1	2	1	1	1	0
Lymphopenia	13	10	4	8*	7	1	2	1
Non-hematological toxicity, number of cases								
AST/ALT elevation	12	0	4	0	6	0	2	0
Interstitial pneumonitis	0	1^c^	0	1^c^	0	0	0	0
Colitis	0	1^d^	0	0	0	0	0	1^d^
Anaphylaxis	0	2^e^	0	0	0	2^e^	0	0
Hemorrhage	1^f^	1^g^	1^f^	0	0	1^g^	0	0

*Abbreviations CRC* colorectal cancer, *LC* lung cancer

^a^NCI-CTC: National Cancer Institute Common Toxicity Criteria

^b^In two of 15 patients, laboratory data was lost

^c^Death caused by pemetrexed-induced pneumonitis

^d^Pseudomembranous colitis due to *Clostridium difficile* infection

^e^Oxaliplatin- and cetuximab-induced anaphylaxis

^f^Nasal bleeding

^g^Hemoptysis

*: *P* < 0.005

One CRC patient experienced Grade 3 hemoptysis and another 2 experienced Grade 3 anaphylaxis (oxaliplatin-induced [*n* = 1] and cetuximab-induced [*n* = 1]). One patient with CCA and GC experienced Grade 3 pseudomembranous colitis as a result of a *Clostridium difficile* infection. One LC patient died of pemetrexed-induced pneumonitis (Grade 5).

### Overall survival

**Fig. 2:**
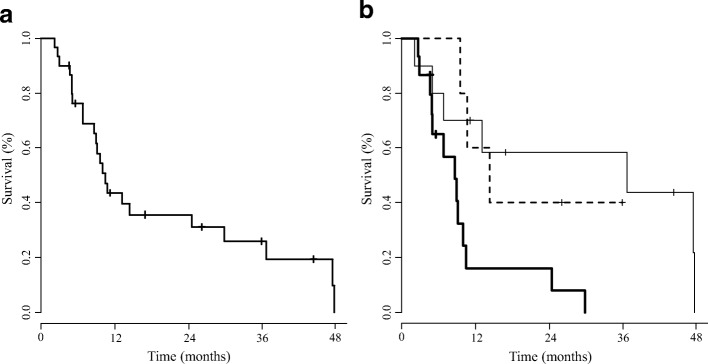
(**a)** Kaplan-Meier curve of overall survival for 30 cancer patients with active *Mycobacterium tuberculosis* who received concurrent chemotherapy between January 1, 2006 and February 29, 2016. The median OS in all patients was 10.5 (8.7–36.7; 95.0% confidence interval) months (**b**) Kaplan-Meier curves of overall survival according to cancer type. Lung Cancer patients are represented by the thick solid line, Colorectal Cancer patients are represented by the thin solid line, and patients with Other malignancies, are represented by the dashed line. The median OS according to the type of malignancy were 8.7 (4.7–10.0), 36.7 (minimum 2.2), and 14.4 (minimum 9.6) months for Lung Cancer, Colorectal Cancer, and Other malignancies, respectively

The median OS in all patients was 10.5 (8.7–36.7; 95.0% confidence interval) months (Fig. 2a). The median OS according to the type of malignancy were 8.7 (4.7–10.0), 36.7 (minimum 2.2), and 14.4 (minimum 9.6) months for LC, CRC, and other malignancies, respectively (Fig. 2b).

## Discussion

This study demonstrates the efficacy and safety of concurrent anti-CCT and anti-*MTB* chemotherapy for cancer patients with active *MTB,* confirming the findings of our previous preliminary study [2] in a bigger sample size with a variety of cancer types.

In Japan, the national rates for success, failed, died, and lost to follow-up of anti-*MTB* chemotherapy in 2015 were 52.8, 0.4, 17.0, and 5.6%, respectively [18]. In Nagoya which is almost similar to Osaka, where our institution is located, the rates of success, failed, died, and the others were 52.0, 0.7, 20.8, and 26.5%, respectively. [19] The success rate of anti-*MTB* chemotherapy in this study may have been higher than previous studies [18, 19], possibly because of no loss to follow-up and no unevaluated patient in the present study. However, it is important that there were no *MTB*-related treatment failures in our study, suggesting that cancer patients with active *MTB* would be able to tolerate anti-*MTB* chemotherapy while receiving anti-CCT as well as non-cancer patients with active *MTB*.

Conversely, the ORRs for all patients combined, LC patients, CRC patients, and other malignancy patients were 36.7, 33.3, 40.0, and 40.0%, respectively.

In 15 LC patients, all 5 patients with SCLC received intensive cytotoxic treatment (carboplatin plus etoposide) as first line chemotherapy (Table 3) with a high response rate (80%), which is similar to the response rate (73%) of similar treatment regimen in the elderly patient or those at poor risk with extensive disease [20]. On the other hand, 10% of response rate in 10 NSCLC patients seems to be low. This may be because 6 (60.0%) of 10 NSCLC patients had received second-line or later anti-CCT after starting anti-*MTB* chemotherapy.

ORR in CRC patients with *MTB* was 40% which was similar to other 1st line combined chemotherapy regimens without molecular targeted therapy in previous reports [21, 22].

Although there were no significant differences in patient characteristics between the LC, CRC, and other malignancy groups, the diagnosis of *MTB* was more frequently reached during chemotherapy in LC patients when compared to patients with other types of malignancies. If LC and pulmonary *MTB* coexist in the lungs, a diagnosis of *MTB* becomes more difficult. Therefore, *MTB* diagnosis tends to be delayed in these patients.

In a Japanese retrospective study, [23] 247 (28.7%) of 861 patients who received *MTB* treatment experienced adverse events. The frequency of adverse events with *MTB* treatment alone, prior to the initiation of concurrent chemotherapy, in our study was equal to that of the a previous study. [23] Besides, upon starting concurrent chemotherapy in LC and CRC, we did not expect any differences in the prevalence of adverse events between this study and previous studies [20–22] except for liver dysfunction which may be correlated with *MTB* treatment.

Concurrent chemotherapy-related toxicities were generally acceptable except death caused by pemetrexed-induced pneumonitis. Grade 3 lymphopenia and higher were significantly more frequent in LC compared to other malignancies. The treatment related lymphopenia may affect poor prognosis in LC patients through decreased immune system shown in previous study [24].

Increased multi-drug resistance and extensive drug resistance among strains of *MTB* is becoming a serious problem worldwide. Hattori et al. reported that 171 patients (0.2%) in Japan were diagnosed with multi-drug resistant *MTB*, and 48 of these (28.1%) were foreigners [25]. Although the number of multi-drug resistant *MTB* cases in Japan is low, Osaka city and Osaka prefecture have the highest prevalence of tuberculosis in Japan (34.4 and 18.2 per 100,000 individuals in 2015, respectively) [26]. Hence, we are exceedingly cautious of an increase in multi-drug resistant *MTB*. In our institution, anti-CCT was suspended as far as possible until the results of the sensitivity test were known.

Given the benefits of a short duration from *MTB* diagnosis to the turnaround of results from sensitivity test, MGIT [5] is recommended for culturing mycobacterial organisms.

In this study, 2 (8.0%) of 25 patients had resistant *MTB* to H and streptomycin and H and Z, respectively, but there was no patient with multi-drug resistant *MTB*. In National-wide survey in Japan [27], the frequencies of drug-resistant isolates from new cases were as follows 8.5% to any drug which was similar to this study.

Poor PS, extensively drug-resistant *MTB (XDR)*, and severe organ dysfunction were the basic contra-indications for concurrent chemotherapy. However, targeted therapy including EGFR-TKI may be used in selected patients despite a poor PS. Furthermore, concurrent chemotherapy may be initiated in patients with rapidly progressive cancer without waiting the results of *MTB* sensitivity test, even if the *MTB* is later categorized as *XDR*. Therefore, there may be no strict contra-indications for concurrent chemotherapy. However, since 6 (20%) of 30 patients experienced adverse events while receiving *MTB* treatment alone until concurrent chemotherapy, awaiting the result of sensitivity test will be important not only for excluding *XDR* but also to evaluate the adverse events of *MTB* treatment alone.

Concurrent use of R, which induces CYP3A4 and CYP2C8 [28, 29], may weaken the clinical efficacy of some anti-CCT agents. In clinical practice, we attempt to not administer concurrent chemotherapy along with Erlotinib, Irinotecan, or Pclitaxel and HRE regimen as much as possible. Therefore, a better choice would be X-based regimen or other regimens without rifampicin.

The limitations of this study include its retrospective design and relatively small sample size. It remains unclear whether the findings of this study could be generalized for hematological malignancies or solid malignancies except LC and CRC, or expanded to other institutions. Therefore, a prospective multi-institutional study to evaluate the efficacy and safety of concurrent anti-CCT and anti-*MTB* chemotherapy in patients with various types of solid tumors and an active *MTB* infection is warranted.

## Conclusions

Concurrent anti-CCT and anti-*MTB* chemotherapy is effective and safe for treating cancer patients with active *MTB.*

## Abbreviations

Anti-CCT: Anti-cancer chemotherapy
ATS: American Thoracic Society
CC: Concurrent chemotherapy
CCA: Cholangiocellular carcinoma
CCT: Cancer chemotherapy
CI: Confidence interval
CR: Complete response
CRC: Colorectal cancer
CT: Computed tomography
E: Ethambutol
GC: Gastric cancer
H: Isoniazid
IDSA: Infectious Diseases Society of America
LAMP: Loop-mediated isothermal amplification
LC: Lung cancer
MDR-TB: Multi-drug resistant *Mycobacterium Tuberculosis*
MGIT: Mycobacteria Growth Indicator Tube
MST: Median survival time
*MTB*: *Mycobacterium tuberculosis*
NE: Not evaluable
NSCLC: Non-small cell lung carcinoma
PCR: Polymerase chain reaction
PD: Progressive disease
PR: Partial response
*XDR*: Extensively drug-resistant *MTB*
Z: Pyrazinamide
